# Dissociation between cortical and spinal excitability of the antagonist muscle during combined motor imagery and action observation

**DOI:** 10.1038/s41598-019-49456-8

**Published:** 2019-09-11

**Authors:** Toshiyuki Aoyama, Fuminari Kaneko, Yukari Ohashi, Yutaka Kohno

**Affiliations:** 1 Department of Physical Therapy, Ibaraki Prefectural University of Health Sciences, 4669-2 Ami, Ami-Machi, Inashiki-gun, Ibaraki, 300-0394 Japan; 2 Department of Rehabilitation Medicine, Keio University School of Medicine, 35 Shinanomachi, Shinjuku-ku, Tokyo 160-8582 Japan; 3 Center for Medical Sciences, Ibaraki Prefectural University of Health Sciences, 4669-2 Ami, Ami-Machi, Inashiki-gun, Ibaraki, 300-0394 Japan

**Keywords:** Motor cortex, Neurophysiology

## Abstract

Inhibitory neural control of antagonist muscle is one of the fundamental neural mechanism of coordinated human limb movement. Previous studies have revealed that motor execution (ME) and motor imagery (MI) share many common neural substrates; however, whether inhibitory neural activity occurs during MI remains unknown. In addition, recent studies have demonstrated that a combined MI and action observation (MI + AO) produces strong neurophysiological changes compared with MI or AO alone. Therefore, we investigated inhibitory changes in cortical and spinal excitability of the antagonist muscle during MI + AO and ME. Single-pulse transcranial magnetic stimulation (TMS) experiments revealed that corticospinal excitability of the antagonist muscle was decreased during MI + AO. Conversely, F-wave experiments showed that F-wave persistence of the antagonist muscle increased. Paired-pulse TMS experiment also demonstrated that short-interval intracortical inhibition (SICI) did not contribute to this inhibition. Therefore, cortical mediated inhibition, except for SICI, may be related to this inhibition. Conversely, such clear inhibition of the antagonist muscle was not observed during ME, presumably owing to the effects of muscle contraction to decelerate the movements and/or sensory input accompanying the joint movements. These findings provide important insights into the neurophysiological differences between MI + AO and ME.

## Introduction

Motor imagery (MI) is defined as the mental simulation of a given movement that is internally reproduced within brain without any muscular output^1,2^. Previous studies using functional imaging have demonstrated that motor execution (ME) and MI share many common neural substrates, including the primary motor cortex, supplementary motor area, premotor cortex, parietal cortex, and cerebellum^3^. TMS studies have also revealed that the corticospinal excitability of the prime mover of the imagined movement increases during MI^4–9^. However, little is known regarding the effect of MI on the non-prime mover, such as the antagonist muscle.

Usually, MEP amplitude obtained from the agonist muscle increases before the movement onset^10^. Since this corticospinal excitability change is not accompanied by an increase in agonist EMG activity, as well as during MI, the neurophysiological changes during MI and before movement onset are similar. On the other hand, a previous study revealed that the corticospinal excitability of the antagonist muscle is suppressed before movement onset^11^. Therefore, considering such neurophysiological similarities occurring during MI and before movement onset in the agonist muscle, we hypothesized that corticospinal excitability of the antagonist muscles would be suppressed during MI. However, no study has reported a significant reduction in MEP amplitudes of the antagonist muscle during MI^6,8,12,13^.

Recent studies have examined the effect of MI during action observation (AO). At the behavior level, MI during AO (MI + AO) task has more potential to improve motor function, such as muscle strength or upper limb function, in stroke patients compared with MI or AO alone^14,15^. Other studies using fMRI have revealed higher brain activity in MI + AO than AO or MI alone^16–20^. In addition, TMS studies have demonstrated that MEP amplitude increases in MI + AO in agonist muscles of the imagined movement compared with during AO or MI alone^21,22^. However, the effects of MI + AO on the antagonist muscle have not been examined. Taking into account the potent effect of MI on neurophysiological changes during AO, we assumed that the corticospinal excitability of the antagonist muscle is suppressed by MI + AO, as it was observed before the onset of ME. Therefore, the primary aim of the present study was to evaluate inhibitory neuronal activity in the antagonist muscle during MI + AO.

Unlike MI + AO, which is not accompanied by actual muscular output and joint movements, the antagonist muscle begins to activate before the middle of the full range of movement to decelerate joint movement during ME^23,24^. Because there is a strong positive correlation between EMG activity and MEP amplitude^25^, corticospinal excitability in antagonistic muscles during the middle phase of ME is predicted to increase. Therefore, we assume that there are clear differences in corticospinal and spinal excitability changes between during ME and MI + AO. Taking this into account, the secondary aim of the present study was to examine the neurophysiological similarities and differences between ME and MI + AO.

To test our hypotheses, we conducted the following five experiments. In Experiment 1, corticospinal excitability of the index finger abductor muscle (first dorsal interosseous, FDI) during MI + AO of index finger abduction-adduction movement was examined using single-pulse TMS. In Experiment 2, we conducted an F-wave study to examine the contribution of spinal excitability to corticospinal excitability changes. In Experiment 3, paired-pulse TMS experiments were performed to explore the involvement of short-interval intracortical inhibition (SICI) in corticospinal excitability changes. In Experiment 4, corticospinal excitability during ME was tested to evaluate neurophysiological similarities and differences between ME and MI + AO. Finally, in Experiment 5, we conducted an experiment of the SICI paradigm during ME.

Based on previous findings that neurons controlling the agonist and antagonist muscles in the primary motor cortex have strong neuronal interconnections^26,27^, we hypothesized that intracortical inhibition, particularly for SICI, which plays a crucial role in selective finger movement^28–30^, contributes to inhibition in corticospinal excitability during MI + AO.

## Methods

### Subjects

The subjects were all right-handed according to the Edinburgh handedness inventory. All subjects provided written informed consent before participating in this experiment. The experimental protocol was approved by the ethics committee of the Ibaraki Prefectural University of Health Sciences. All procedures conformed to the standards set out in the World Medical Association Declaration of Helsinki.

### MI + AO task

**Figure 1:**
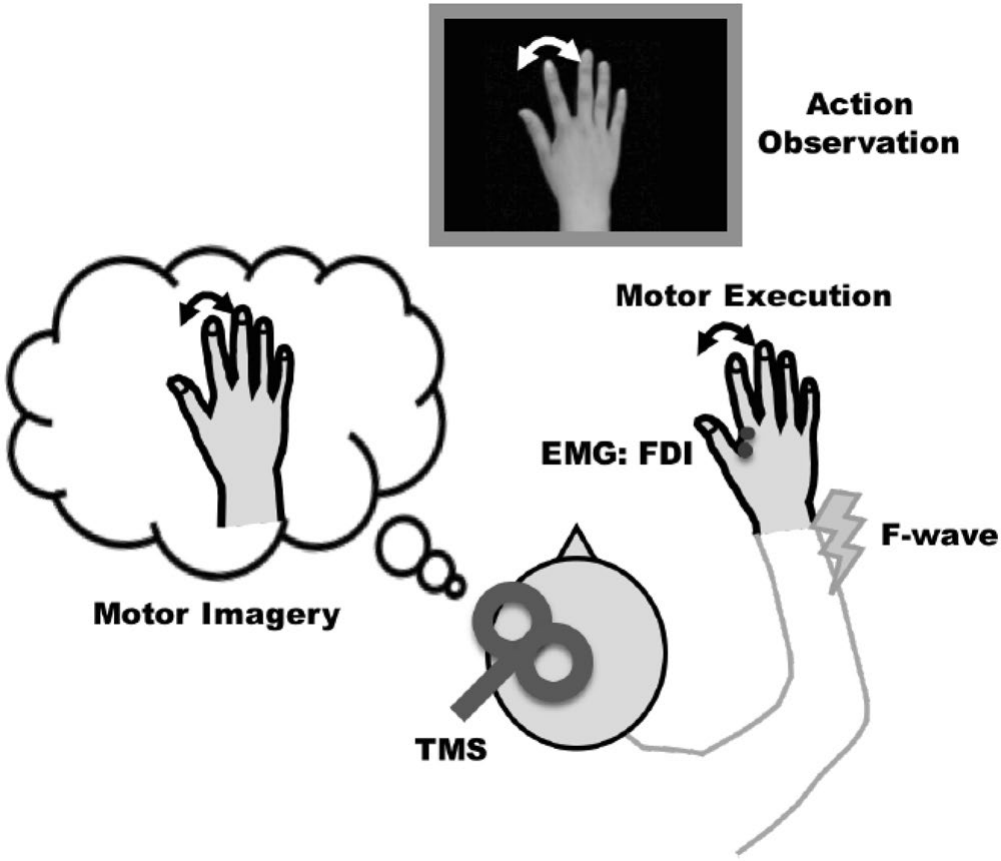
Schematic illustration of the experimental setup in Experiments 1–5. The subjects performed motor imagery (Experiments 1–3) or motor execution task (Experiment 4, 5) of index finger movement while they observed the hand movement. EMG signals were recorded from the FDI muscle. TMS was applied at the left motor cortex in Experiments 1, 4 (single-pulse), and 3, 5 (paired-pulse). In Experiment 2, electrical stimulation was applied to the ulnar nerve at the wrist to induce F-waves.

**Figure 2:**
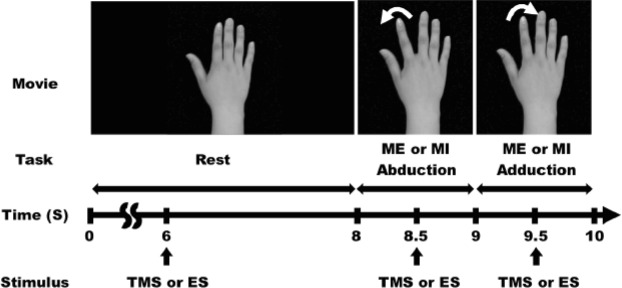
Schematic diagram of the time course of the task and stimulus. The movie showed the static hand image (8 s), right index finger abduction (1 s), and adduction (1 s). The subject performed motor imagery (MI) or motor execution (ME) task, concurrently with the index finger movement in the movie. TMS (Experiments 1, 3, 4, 5) or electrical stimulation (Experiment 2) was applied 6 s after the beginning of the rest phase or 0.5 s after the beginning of index finger abduction and adduction.

Schematic illustration of the experimental setup is shown in Fig. 1. The participants were seated comfortably in a chair with their right hand lying relaxed on a side table. The 10-sec movie, which consisted of three phases: the static image of the hand (rest, 8 s), right index finger abduction (1 s), and adduction (1 s) movement, was repeatedly displayed on a computer screen that was placed in front of the subjects (Fig. 2). The subjects were instructed to imagine the kinesthetic sensation generated by the actual index finger abduction–adduction movement (kinesthetic MI) synchronously with the index finger movement projected on the computer screen^14,31^. The subjects practiced this without any overt muscle contraction using electromyographic feedback from the FDI muscle. The Kinesthetic and Visual Imagery Questionnaire (KVIQ) was used to evaluate the MI ability of the participants^32^.

### Electromyography

Before electrode attachment, the skin was rubbed with alcohol and abraded with abrasive skin prepping gel. Surface Ag-AgCl electrodes were placed over the FDI muscle in a belly-tendon montage. The FDI works as an agonist during index finger abduction, whereas it works as an antagonist during index finger adduction. Electromyography (EMG) signals were amplified (Neuropack MEB2300; Nihon Kohden, Japan) at an appropriate level, and band-pass filtered at 5 Hz–5 kHz. All signals were sampled at 10 kHz and stored in a laboratory computer for offline analysis.

### Experiment 1: single-pulse TMS experiment (MI + AO task)

Thirteen healthy adults (mean ± SD, 20.6 ± 0.7 years) participated in this experiment. A Magstim 2002 stimulator (Magstim Co., Whitland, UK) connected to a figure-of-eight coil (diameter of each loop = 70 mm) was used to elicit MEPs from the right FDI muscle. The handle of the coil was positioned pointing backward and laterally at a 45° angle from the midline to induce anteromedial current direction in the left brain, and to activate the corticospinal tract transsynaptically^33,34^. The hot spot is defined as the optimal scalp site for eliciting the most consistent MEP amplitude from the muscle, with stimulus intensity marginally above the threshold. The resting motor threshold (RMT) was defined as the lowest intensity that produced MEP amplitude of >50 μV in at least 5 out of 10 trials. In the test trials, a stimulus intensity of 140%RMT was used. Single-pulse TMS was applied at rest (6 s after the beginning of the rest phase) and the middle phase of the index finger abduction or adduction (0.5 s after the beginning of each movement, Fig. 2). To avoid prediction of the stimulus timing, TMS was delivered in a randomized order using the LabVIEW program (National Instruments, USA). The inter-trial interval of TMS varied with the timing of the previous and subsequent stimulus (6.5–13.5 s). At least 14 MEPs were recorded during each phase of the MI + AO. All trials in which the participant contracted the FDI muscle involuntary were rejected and another trial was recorded. After recording, we reconfirmed the EMG silence by offline analysis. If any FDI muscle activity 0–50 ms prior to TMS was visually detected, the trial was excluded for further analysis. One-way repeated measures analysis of variance (ANOVA) was used to test the effect of the phase (rest, index finger abduction, and adduction) on MEP amplitude and average rectified value (ARV) of the background EMG activity (0–50 ms prior to stimulation). Multiple comparisons tests were made using the Bonferroni correction, with the level of significance set at p < 0.05.

### Experiment 2: F-wave experiment (MI + AO task)

Fifteen healthy adults (mean ± SD, 21.4 ± 1.5 years) participated in this experiment. F-waves were recorded from the FDI muscle. Supramaximal electrical stimulation with a rectangular electrical pulse of 0.2 ms duration was applied to the ulnar nerve at the wrist (Neuropack MEB2300; Nihon Kohden, Japan). Similar to Experiment 1, the Lab VIEW program (National Instruments, USA) was used to apply electrical stimulation during the rest period (6 s after the beginning of the rest phase), the middle phase of the index finger abduction or adduction (0.5 s after the beginning of each movement) in a randomized order (Fig. 2). The inter-trial interval of electrical stimulation varied with the timing of the previous and subsequent stimulus (6.5–13.5 s). At least 30 F-waves were recorded during each of the three phases. All trials in which the participant contracted the FDI muscle involuntary during MI + AO were rejected and another trial was recorded. After recording, we reconfirmed the EMG silence by offline analysis. If any FDI muscle activity 0–50 ms prior to electrical stimulation was visually detected, the trial was excluded from further analysis. F-wave persistence was defined as the number of measurable F-wave responses divided by all trials of supramaximal stimulation^35^. Furthermore, the F/M ratio (F-wave amplitude divided by M-wave amplitude, %) was calculated. One-way repeated measures ANOVA was used to test the effect of the phase on F-wave persistence, F/M ratio, and ARV of background EMG activity (0–50 ms prior to stimulation). Multiple comparisons were made using the Bonferroni correction, with the level of significance set at p < 0.05.

### Experiment 3: paired-pulse TMS experiment (MI + AO task)

Thirteen healthy adults (mean ± SD, 21.3 ± 0.6 years) participated in this experiment. A Magstim 2002 stimulator (Magstim Co., Whitland, UK) connected to a figure-of-eight coil (diameter of each loop = 70 mm) was used to elicit MEPs from the right FDI muscle. The handle of the coil was positioned pointing backward and laterally at a 45° angle from the midline to induce anteromedial current direction in the left brain and to activate the corticospinal tract transsynaptically. The hot spot is defined as the optimal scalp site for eliciting the most consistent MEP amplitude from the muscle, with stimulus intensity marginally above the threshold. Paired-pulse TMS with an inter-stimulus interval of 2 ms was used to examine SICI. The test stimulus intensity was adjusted to produce an average MEP of approximately 1.0 mV in each phase of the MI + AO. The conditioning stimulus intensity of each phase of MI + AO was set at a level producing approximately 30% inhibition of the test MEP amplitude at rest to avoid the floor effect. Active motor threshold (AMT) was defined as the minimum stimulus intensity producing a peak-to-peak MEP amplitude >200 μV in at least five of the 10 consecutive trials during 10% maximum voluntary contraction of the index finger abduction. After recording AMT, the conditioning stimulus intensity was set to 70% AMT under resting conditions. If the level of inhibition was <30%, the conditioning stimulus intensity was increased by 5% AMT. Similar to Experiments 1 and 2, the Lab VIEW program (National Instruments, USA) was used to apply the TMS during rest period (6 s after the beginning of the rest phase), the middle phase of the index finger abduction or adduction (0.5 s after the beginning of each movement) in a randomized order (Fig. 2). The inter-trial interval of TMS varied with the timing of the previous and subsequent stimulus (6.5–13.5 s). A minimum of 12 single and paired pulse MEPs were recorded during each phase of the MI + AO. All trials in which the participant contracted the FDI muscle involuntary were rejected and another trial was recorded. After recording, we reconfirmed the EMG silence by offline analysis. If any FDI muscle activity 0–50 ms prior to TMS was visually detected, the trial was excluded for further analysis. Averaged conditioned MEP amplitude was divided by the averaged test MEP amplitude of the same condition (phase). One-way repeated measures ANOVA was used to test the effect of the phase factor on conditioned MEP (% test pulse alone), test pulse MEP amplitude, and the test pulse stimulus intensity. Two-way repeated measures ANOVA was used to test the phase factor and stimulation factor (single-pulse, paired-pulse) on the ARV of the background EMG activity (0–50 ms prior to stimulation). Multiple comparisons using the Bonferroni method were made, and the level of significance was set at p < 0.05.

### Experiment 4: single-pulse TMS experiment (ME task)

Thirteen healthy adults (mean ± SD, 20.6 ± 0.7 years) participated in this experiment. The experimental procedure except for the task was same as Experiment 1. In this experiment, the subjects moved their right index finger while they were watching the same movie as Experiments 1–3. Prior to recording, they practiced the task until they could move their index finger synchronously with the movie^14,31^ while confirming there was no electromyographic activity during the rest phase. They were also instructed to practice the index finger abduction–adduction movement as selectively as possible. Single-pulse TMS was applied at rest (6 s after the beginning of the rest phase) and the middle phase of the index finger abduction or adduction (0.5 s after the beginning of each movement, Fig. 2). One-way repeated measures ANOVA was used to test the effect of the phase (rest, index finger abduction, and adduction) on MEP amplitude and ARV of the background EMG activity (0–50 ms prior to stimulation). Multiple comparisons tests were made using the Bonferroni correction, with the level of significance set at p < 0.05.

### Experiment 5: paired-pulse TMS experiment (ME task)

Seven healthy adults (mean ± SD, 21.1 ± 0.7 years) participated in this experiment. The experimental procedure except for the task was the same as Experiment 3. The ME task was the same as Experiment 4.

## Results

### Experiment 1: Corticospinal excitability changes during MI + AO

**Figure 3:**
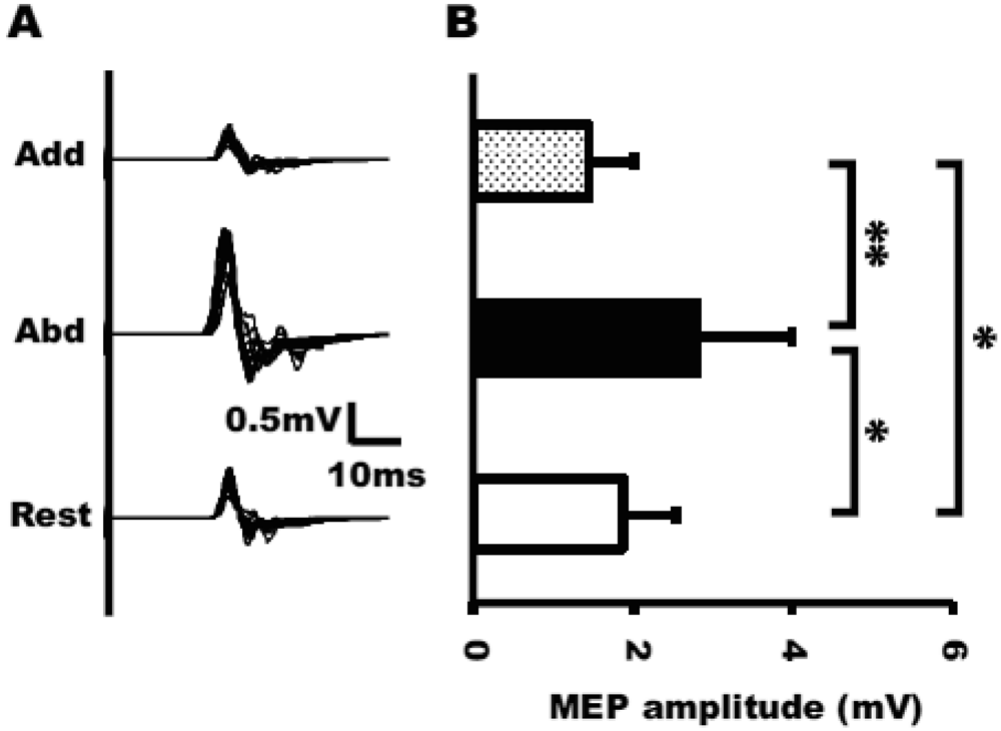
Superimposed raw MEP amplitude for a representative subject recorded from the FDI muscle during MI + AO of index finger abduction (abd), adduction (add), and rest phases (**A**). Average ± SD of the FDI MEP amplitude during MI of three phases (**B**). (*p < 0.05; **p < 0.01).

Superimposed FDI MEP raw data for a representative subject is shown in Fig. 3A. Figure 3B shows the average (±SD) FDI MEP amplitude for each phase during MI + AO. There was a significant main effect of phase (stimulus timing) on FDI MEP amplitude [F (2, 24) = 13.63, p < 0.0005]. Bonferroni’s multiple comparison test revealed that there was a significant inhibitory effect on the FDI MEP amplitude in the adduction (1.43 ± 0.56 mV) phase compared with the rest phase (1.87 ± 0.66 mV). FDI MEP amplitude in the abduction (2.78 ± 1.20 mV) phase was significantly larger than that in the rest and adduction phases. There was no significant main effect of phase on background EMG activity in FDI during MI + AO [F (2, 24) = 0.24, p = 0.79, rest; 3.21 ± 1.47 μV, abduction; 3.25 ± 1.59 μV, adduction; 3.26 ± 1.44 μV].

The average (±SD) KVIQ scores in Experiment 1 was 40.1 (±6.0).

### Experiment 2: Spinal excitability changes during MI + AO

**Figure 4:**
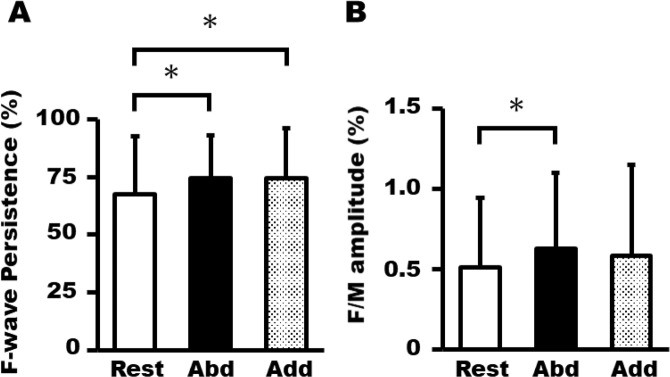
Average ± SD of F-wave persistence (**A**) and F/M amplitude (**B**) from the FDI muscle during MI + AO of abduction (abd), adduction (add), and rest phases. (*p < 0.05).

Figure 4 presents the average (±SD) FDI F-wave persistence and F/M amplitude for each phase during MI + AO. One-way repeated measures ANOVA revealed that there was a significant main effect of phase on FDI F-wave persistence [F (2, 28) = 6.05, p = 0.007]. FDI F-wave persistence was significantly larger in the abduction (74.6% ± 18.6%) and adduction phases (74.4% ± 21.8%) than in the rest phase (67.6% ± 25.1%). The FDI F/M ratio was significantly larger in the abduction phase than in the rest phase (rest; 0.51% ± 0.43%, abduction; 0.63% ± 0.47%, adduction; 0.58% ± 0.57%). There was no significant main effect of phase on background EMG activity in FDI [F (2, 28) = 1.47, p = 0.246, rest; 2.47 ± 0.79 μV, abduction; 2.56 ± 0.60 μV, and adduction; 2.36 ± 0.64 μV].

The average (±SD) KVIQ scores in Experiment 2 was 44.1 (±4.3).

### Experiment 3: SICI changes during MI + AO

**Figure 5:**
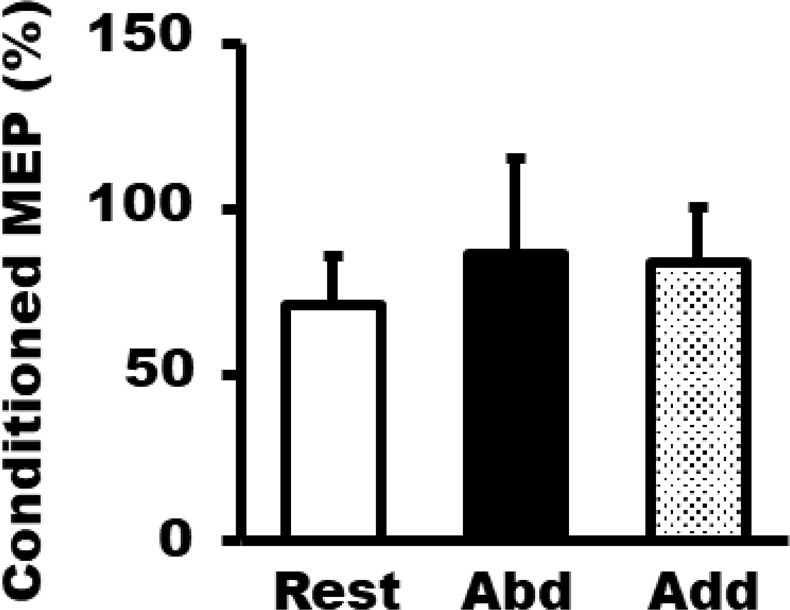
Average ± SD of conditioned MEP (% test pulse alone) from the FDI muscle during MI + AO of abduction (abd), adduction (add), and rest phases.

Figure 5 shows the average (±SD) FDI conditioned MEP (% test pulse alone) amplitude during MI + AO. There was no significant effect of phase on test pulse FDI MEP amplitude [F (2, 24) = 0.025, p = 0.975, rest; 0.97 ± 0.18 mV, abduction; 0.96 ± 0.21 mV, and adduction; 0.96 ± 0.19 mV]. The test pulse stimulus intensity (61.9% ± 9.5%) was significantly smaller in the abduction phase than in the rest (66.1% ± 10.3%) and adduction phases [70.2% ± 12.4%, F (2, 24) = 22.95, p < 0.0005]. The average (±SD) conditioning stimulus intensity of the three phases was 84.2% ± 7.3% AMT. One-way repeated measures ANOVA revealed no significant main effect [F (2, 24) = 2.01, p = 0.16, rest; 70.8% ± 14.6%, abduction; 86.4% ± 29.1%, adduction; 83.6% ± 17.0%] of phase on conditioned MEP. For the background EMG of FDI muscle, there was no significant interaction [F (2, 12) = 2.77, p = 0.10] between phase and stimulation factors [F (2, 24) = 1.15, p = 0.33]. There was also no significant main effect of phase [F (2, 24) = 1.54, p = 0.24] and stimulation [F (1, 12) = 0.755, p = 0.402] factor on the FDI background EMG.

The average (±SD) KVIQ scores in Experiment 3 was 44.2 (±4.5).

### Experiment 4: corticospinal excitability changes during ME

**Figure 6:**
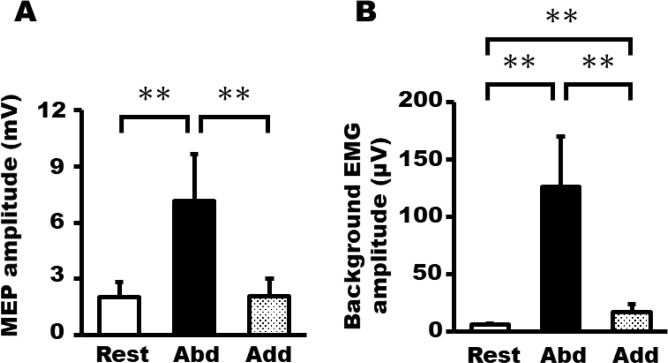
Average ± SD of the FDI MEP amplitude (**A**) and background EMG activity (**B**) during ME of three phases. (**p < 0.01).

Figure 6 shows the average (±SD) FDI MEP amplitude and background EMG activity for each phase during ME. One-way repeated measures ANOVA revealed a significant main effect of phase on FDI MEP amplitude [F (2, 24) = 50.47, p < 0.0005] and background EMG signals [F (2, 24) = 83.61, p < 0.0005] during ME. Bonferroni’s multiple comparison test revealed that FDI MEP amplitude was significantly larger in the abduction phase (7.13 ± 2.50 mV) than in the rest (2.01 ± 0.81 mV) and adduction phases (2.09 ± 0.91 mV). FDI background EMG activity was significantly larger in the abduction phase (126.08 ± 44.02 μV) during ME than in the rest (6.37 ± 0.43 μV) and adduction phases (16.99 ± 7.15 μV). Furthermore, FDI background EMG activity was significantly larger in the adduction phase than in the rest phase.

### Experiment 5: SICI changes during ME

**Figure 7:**
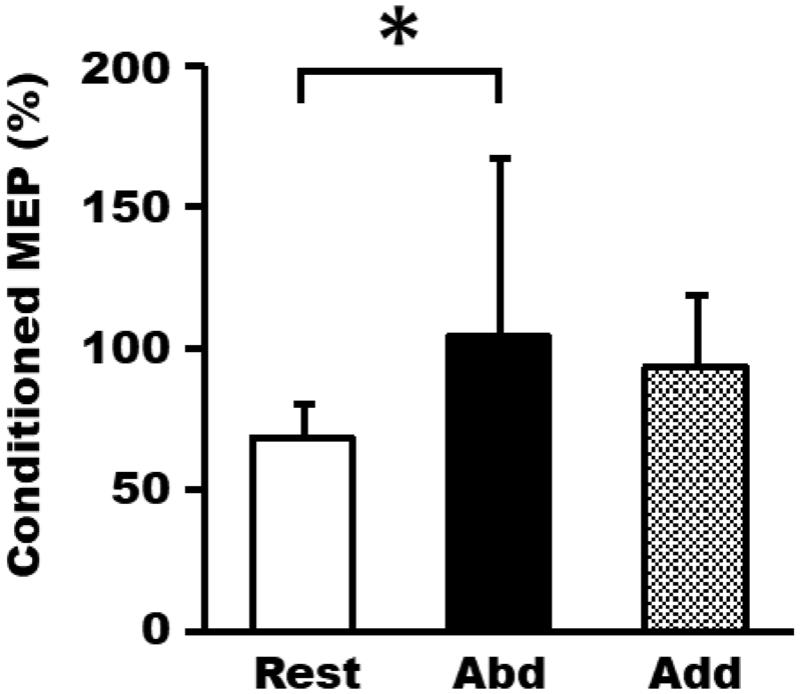
Average ± SD of conditioned MEP (% test pulse alone) from the FDI muscle during ME of abduction (abd), adduction (add), and rest phases. (*p < 0.05).

There was no significant main effect of phase on the test MEP amplitude of the FDI muscle during ME (F [2, 12] = 2.94, p = 0.09, rest; 0.98 ± 0.07 mV, abduction; 1.06 ± 0.16 mV, adduction; 0.86 ± 0.21 mV). The test stimulus intensity in the abduction phase (39.3% ± 5.9%) was significantly lower than that in the rest (58.6% ± 8.2%) and adduction phases (58.4% ± 10.1%, F [2, 12] = 64.2, p < 0.0005). The average (±SD) conditioning stimulus intensity of the three phases was 78.5% ± 3.8% AMT. One-way repeated measures ANOVA revealed a significant main effect (F [2, 12] = 6.20, p < 0.014, Fig. 7) of phase on the conditioned MEP amplitude (% test pulse alone). The conditioned MEP in the abduction phase (104.1% ± 24.3%) was significantly larger than that in the rest phase (68.0% ± 14.2%). In addition, there was a trend in the adduction phase (93.8% ± 25.1%) for the FDI-conditioned MEP amplitude to be larger than that in the rest phase, although statistical significance was not reached (p = 0.072). For the background EMG of the FDI muscle, there was no significant interaction (F [2, 12] = 0.64, p = 0.55) between the phase and stimulation (single-pulse, paired-pulse) factors. There was a significant main effect of phase factor on FDI background EMG (F [2, 12] = 36.80, p < 0.0005). Background EMG in the abduction and adduction phases was significantly larger than that in the rest phase. No significant main effect of the stimulation factor on FDI background EMG (F [1, 6] = 0.31, p = 0.60) was detected.

## Discussion

The most striking finding of this study was the clear inhibitory changes in the corticospinal excitability of the antagonist muscle that occurred during MI + AO. However, F-wave experiments showed that spinal excitability increased not only in the agonist muscle, but also in the antagonist muscle during MI + AO. Because corticospinal excitability reflects changes in both cortical and spinal excitability^36^, if cortical excitability changes did not occur, corticospinal excitability should increase owing to increased spinal excitability. However, this is inconsistent with our results. Therefore, these seemingly contradictory results (decreased corticospinal excitability and increased spinal excitability) in the antagonist muscles during MI + AO suggest that the increase in cortical-mediated inhibition simultaneously occurred with the increase in spinal excitability. In addition, paired pulse TMS experiments demonstrated that SICI did not contribute to this inhibition. These results indicated that cortical-mediated inhibition, except SICI, may be related to inhibitory changes in the corticospinal excitability of the antagonist muscle during MI + AO

Similar to previous MI studies^4–9^, an increase in corticospinal excitability of the FDI muscle was observed when the FDI acted as an agonist muscle during MI + AO. Contrary to the agonist muscle, a reduction in the FDI MEP amplitude was found when the FDI acted as an antagonist muscle during MI + AO. This result indicates that corticospinal excitability of the antagonist muscle decreased during MI + AO. Similar to our study, Ikai *et al*.^12^ reported that decreased in antagonist MEP amplitude in some subjects during MI, but this effect was not statistically significant. To our knowledge, this is the first study to demonstrate clear inhibitory changes in corticospinal excitability during MI + AO. Conversely, Kasai *et al*.^6^ reported that the MEP in the hand extensor muscle was increased during MI of hand flexion. Furthermore, other studies have shown no significant change in MEP amplitude in the antagonist muscle during MI^7,8^. One of the possible reasons for this discrepancy is whether AO was added to the MI or not. A previous study showed that AO could enhance the vividness of MI^37^. Furthermore, some recent TMS studies have demonstrated that the facilitatory effect on corticospinal excitability of the agonist muscle was higher in combinations of MI and AO, compared with either MI or AO alone^21,22^. Although it is unclear whether this neurophysiological change is caused by the vividness of MI, there should be an obvious neurophysiological and psychological difference between MI + AO and the MI. Therefore, AO in combination with MI, which was adopted in the present study, may be one of the reasons responsible for the difference between the results of our present study and previous studies. Other factors, such as the type of muscle contraction (isometric or isotonic contraction) and the modality of MI (first person or third person)^38^ may also contribute to this difference. Further research is required to determine the contributing factors to this inhibition in the MI + AO.

Although we obtained these novel findings in Experiment 1, it remained unclear whether cortical or spinal involvement contributed to the observed inhibitory changes in corticospinal excitability because corticospinal excitability reflects both cortical and spinal excitability changes^36^. To this end, we carried out Experiment 2, which was an F-wave study to explore spinal contribution to this inhibition. Surprisingly, our results showed that F-wave persistence increased not only when the FDI muscle works as an agonist but also when it works as an antagonist. This result indicates that spinal excitability of both agonist and antagonist muscles increases during MI + AO. Conversely, a significant increase in FDI F-wave amplitude was detected only in the abduction phase and not in the adduction phase. This suggests that increased spinal excitability caused by MI is more clear in the agonist muscle than the antagonist muscle. Furthermore, the cause of the difference between the results of F-wave persistence and F-wave amplitude of the antagonistic FDI muscle during MI + AO can be attributed to the difference in sensitivity between the two parameters for detecting spinal excitability changes^39^. Previous studies demonstrated that F-wave persistence and amplitude of the agonist muscle did not change during MI^4,38,40–42^. However, the number of F-waves recorded in previous studies was small compared with the present study (in two studies, only 10 F-waves were recorded). Because F-wave has lower sensitivity to detect spinal excitability changes compared with H-reflex, more F-waves should be recorded to obtain reliable data^39^. Therefore, a difference in the number of recorded F-waves may result in such inconsistent results. Only one study reported an increase in F-wave amplitude of the agonist muscle during MI^43^. However, no study has addressed F-wave of the antagonist muscle during MI or MI + AO. Collectively, the present findings of an increase in FDI F-wave persistence during both index finger abduction and adduction suggest that spinal excitability changes do not appear to contribute to the inhibitory changes in corticospinal excitability, whereas cortical involvement may contribute to it.

Using paired-pulse TMS, we assessed whether inhibitory changes in corticospinal excitability was mediated by SICI in Experiment 3. The results indicated that SICI recorded from the FDI muscle remained unchanged during MI + AO of index finger abduction. Although SICI during MI + AO has not been investigated, some previous research reported SICI in an agonist muscle during pure MI^9,41,44^. Consistent with our results, some previous studies revealed that SICI in an agonist muscle was not modulated during pure MI^9,45^. However, some researchers reported that SICI was decreased during MI^9,41,44^. Stinear *et al*.^9^ proposed that the types of imagined movement (phasic or tonic) or the conditioning stimulus intensity may be associated with these different results. Contrary to our prediction, an increase in SICI was not observed when the FDI muscle acted as antagonist during MI + AO. Accordingly, SICI is not a candidate mechanism of inhibitory changes in corticospinal excitability. To our knowledge, this is the first study to explore SICI of the antagonist muscle during MI + AO.

Considering the findings obtained from Experiments 1, 2, and 3, cortical-mediated inhibition, except SICI, may involve inhibitory changes in corticospinal excitability. There are at least two possible explanations for this mechanism. First, it was reported in a non-human study, that motor cortical zones controlling antagonistic wrist muscles are intrinsically interconnected with zones that control agonist wrist muscles^26,27^. Furthermore, the injection of the GABA(A) antagonist bicuculline methiodide into the primate motor cortex induced unstable manual motor performance caused by the co-contraction of agonist and antagonist muscles^46^. Summarizing the above findings, the inhibitory interconnection between cortical neurons controlling the agonist and antagonist muscles may be a fundamental mechanism underlying the cortical-mediated inhibition of antagonist muscle and contribute to coordinated movement. Therefore, this inhibitory interconnection in the motor cortex may be one of the candidate mechanisms that account for the inhibitory changes during MI + AO.

Second, cortical-mediated increases in inhibitory Ia interneuron activity might be responsible for this inhibitory change. There is good evidence that spinal inhibitory interneurons receive descending inputs^47–49^. Furthermore, human H-reflex experiments have demonstrated that reciprocal Ia inhibition of the soleus muscle increased approximately 70–80 ms prior to the onset of concentric ankle dorsiflexion and was accompanied by increased excitability of the agonist (tibialis anterior) muscle^50^. Since the Ia discharges from the agonist muscle increase only after the onset of the agonist muscle contraction^51^, the increase in both Ia inhibitory interneuron activity and alpha motoneuron activity of the agonist muscle prior to the movement should be due to descending control of the cortical origin, independent of the Ia discharge. Considering the neurophysiological similarity between MI and before the onset of ME, the excitability of Ia inhibitory interneurons may increase at the subthreshold level during MI + AO. In addition, the results of this study, and some previous studies, which indicates an increase in alpha motoneuron excitability of the agonist muscle during MI^52–55^, further support the assumption that simultaneous increases in Ia inhibitory interneuron excitability occur with agonist motoneuron excitability.

Regarding the neural mechanisms of ME and MI, many neuroimaging studies have demonstrated that ME and MI share common neural substrates^56–58^. Furthermore, TMS studies have shown that corticospinal excitability of an agonist muscle increases during MI^4–9^ as well as ME. To date, however, little is known about the neurophysiological similarities or differences between ME and MI of the antagonist muscle. Therefore, we examined corticospinal excitability changes during ME using single-pulse TMS in Experiment 4. The present results demonstrated that although the background EMG activity clearly increased, the MEP amplitude remained unchanged in the antagonistic FDI muscle during ME of index finger adduction movement. It is well known that corticospinal excitability changes normally depend on the degree of muscle contraction. Therefore, the contradictory results between background EMG activity and corticospinal excitability changes in the present study indicate that there was no increase in excitability of motor cortical projections to the antagonist muscle and that the change in EMG activity was produced by noncortical mechanisms, such as Ia afferent input to spinal motoneurons and/or activity in non-corticospinal descending pathways^10^. In addition, this effect may be counterbalanced by cortical-mediated inhibition.

The results of Experiment 5 showed no increase (rather, a tendency to decrease) in the SICI in the antagonistic FDI during ME. Only a few studies have examined the SICI in antagonist muscle during ME. Reynolds and Ashby^59^ reported that the SICI in the antagonistic hand muscle did not change during the isometric contraction of the hand flexor or extensor muscle. An important difference between our study and the previous one is the type of muscle contraction. In this study, the isotonic contraction accompanied by joint movement would be expected to increase Ia discharges from the antagonist muscle. Rosenkranz and Rothwell^60^ demonstrated that increased Ia afferent discharge induced by tendon vibration served to attenuate the SICI. Therefore, increases in the Ia discharge from antagonistic FDI muscle during index finger adduction movement might explain the tendency for a decrease in the SICI in our study. In any case, these findings suggest that the SICI does not contribute to the cortical-mediated inhibition of the antagonist muscle during ME.

We did not record the F-wave in this study because it could not be acquired properly during ME owing to the influence of V-wave contamination^61^. A previous study investigating time-course changes in the spinal excitability of the antagonist muscle revealed that the H-reflex amplitude of antagonist muscle decreases before movement onset but increases accompanying the increase in background EMG of the antagonist muscle after movement^10^. Therefore, it seems that the spinal excitability of the antagonist muscle is not suppressed during ME. Taking into consideration the results of Experiments 1–3, which suggest the possibility of cortical-mediated inhibition in an antagonist during MI + AO, similar inhibitory neural activity may occur during ME and MI + AO of the antagonist muscle.

Conversely, the most striking difference between ME and MI + AO of the antagonist muscle was the presence or absence of EMG activity. Some previous studies have shown that antagonist muscle activity is initiated before the middle of the full range of motion to decelerate the movement^23,24^. Therefore, the FDI muscle prepares to decelerate the adduction movement by lengthening contraction at the middle of the adduction phase of ME. For this reason, background EMG activity in FDI during the adduction movement was significantly increased, whereas no background EMG activity was detected during MI + AO. Another important difference between ME and MI + AO of the antagonist muscle was whether actual movements and the accompanying afferent input were present or not. In previous studies focusing on the influence of sensory input accompanying actual movement, corticospinal excitability change during passive limb movement was examined^62–68^. While some studies reported increased corticospinal excitability^65,66^, others reported decreased corticospinal excitability during lengthening passive limb movement^62–64,67,68^. Although the cause of this difference is unknown, factors such as target muscle and/or velocity of passive limb movement may have contributed. In either case, it is likely that sensory inputs accompanying actual movement, particularly from muscle spindles, lead to corticospinal excitability changes. By contrast, because actual movement does not occur during MI + AO, no accompanying sensory input occurs. Collectively, corticospinal excitability changes of the antagonist muscle during MI + AO should occur independently of the effect of lengthening contraction to decelerate finger movement and sensory afferent input. We speculate that inhibitory neural activity similar to MI + AO occurs during ME, but it may be obscured by factors that occur only during ME, such as lengthening contraction and sensory input.

Our study has several limitations. First, EMG activity from the first palmar interosseous muscle, which is the antagonist muscle of FDI, was not recorded. Owing to its anatomical location, it is difficult to suitably record the EMG activity from this muscle. Considering the anatomical distance between the FDI and first palmar interosseous muscle, the effect of cross-talk is likely small^69^. However, the possibility of cross-talk cannot be ruled out. Second, although we obtained novel findings indicating that cortical-mediated inhibition of the antagonist muscle, except for SICI, occurs during MI + AO, we were unable to identify the precise neural mechanisms involved in this inhibition. As described above, we believe that cortical-mediated increases in inhibitory Ia interneuron activity might be responsible for this inhibition. Therefore, we should conduct an H-reflex conditioning-test paradigm experiment to clarify the source of inhibition. Unfortunately, because the H-reflex is difficult to record from the FDI muscle, we should conduct an H-reflex conditioning-test paradigm experiment for other muscles in which the H-reflex can be recorded (e.g., flexor carpi radialis or soleus muscles).

## Conclusion

We examined whether inhibitory neuronal activity of the antagonist muscle occurs during MI + AO of index finger movement using single and paired-pulse TMS and F-wave. Our novel findings indicated that cortical-mediated inhibition, except for SICI, occurs during MI + AO. Furthermore, such clear inhibition of the antagonist muscle was not observed during ME, presumably owing to the effects of muscle contraction and/or sensory input accompanying the joint movements. These findings provide important insights into the neurophysiological similarities and differences between MI + AO and ME. Regarding clinical relevance, because MI + AO can selectively drive the inhibitory neural activity of the antagonist muscles, unlike actual motor execution, MI + AO has potential therapeutic benefits of inducing selective inhibitory neural activity for patients with excessive or non-selective muscle activity that occurs by motor execution (e.g., spasticity or focal hand dystonia).

## Data Availability

Data that support the findings of this study are available from the corresponding authors upon reasonable request.
